# The usability, feasibility and fidelity of the Ethics Quarter e-learning intervention for nurse managers

**DOI:** 10.1186/s12909-022-03241-w

**Published:** 2022-03-15

**Authors:** Laura Laukkanen, Riitta Suhonen, Eliisa Löyttyniemi, Helena Leino-Kilpi

**Affiliations:** 1 Department of Nursing Science, University of Turku, FI-20014 Turku, Finland; 2 Department of Obstetrics and Gynecology, Turku University Hospital, Turku, Finland; 3 Turku University Hospital and the Welfare Division of the City of Turku, University of Turku, FI-20014 Turku, Finland; 4 Department of Biostatistics, University of Turku, FI-20014 Turku, Finland; 5 Turku University Hospital, University of Turku, FI-20014 Turku, Finland

**Keywords:** Ethics, Nursing management, Ethical activity profile of nurse managers

## Abstract

**Background:**

Nurse managers (NMs) expect support to carry out their ethical activities in a complex health care environment. In this study, the Ethics Quarter (EQ) is suggested as a new educational ethics e-learning intervention for nurse managers. The aim of this study was to evaluate the usability, feasibility and fidelity of the EQ. The goal was to create a new way to support NMs’ ethical activity profile (developing one’s own ethics knowledge, influencing ethical issues, conducting or implementing ethics research, identifying and solving ethical problems) for the use of healthcare organizations.

**Methods:**

The EQ was developed under guidance of the criteria for complex interventions in health care (CReDECI2) guideline. A cross-sectional survey was conducted within the intervention group after a randomized controlled trial (the main study is registered in ClinicalTrials.gov with the identifier: 04234503). The participants were NM members of the Union of Health and Social Care Professionals in Finland (*n* = 95).

**Results:**

A system usability scale (SUS) assessed the overall usability of EQ as good (a mean SUS score of 85.40 out of 100). Positive feedback about the EQ’s feasibility was reported in structured and open questions (a good, necessary and practical research knowledge-based e-learning intervention for all nurse managers*)* and recommendations for further development (intervention contents could be even more challenging and interactive) were highlighted. Fidelity, measured with Google Analytics, reported shorter time used by NMs on the EQ education than estimated.

**Conclusions:**

The findings support the high usability, feasibility and average fidelity of the EQ intervention and its potential while also providing evidence for the development of future ethics education. Health care organizations would benefit from adopting the EQ to support the ethical activities and ethical activity profile of NMs. Additionally, this study provides an example of ethics intervention development and evaluation in nursing research.

## Background

Ethics is a fundamentally central, everyday basis of the work of nurse managers (=NMs), referring to all responsible for leadership, management and administrative positions and working as a part of the health care administration at unit, middle, and strategic level management. NMs have a wide impact on their staff, organizations and patients in health care. Thus, it is crucial that NMs fulfill their various ethical responsibilities by performing ethical activities. In this study, these ethical activities of NMs, based on previous literature, have been theoretically outlined, and using deductive reasoning summarized into a new construct, the ethical activity profile of NMs [1], consisting of five dimensions:

Firstly, *NMs have* *the responsibility to develop their own ethics knowledge*. NMs should develop their own skills, competence and knowledge base in ethics throughout their career [2]. In addition to basic education in ethics, NMs need continuing education about values and ethics [2, 3]. Secondly, *NMs have a responsibility to influence ethical issues*. NMs should work as ethics spokespersons and political strategists [4], articulate the application of ethical principles to nursing, integrate values into everyday nursing practice and create an environment with high ethical standards [3, 5, 6], by visibly and intentionally role modeling ethical action [7]. NMs should provide support for the nursing staff in ethically problematic situations [8], create ethical discussions and ethical forums [9, 10] and support the ethical competence development of nurses [11]. Influencing can also materialize in the form of official posts, work groups or committees. Thirdly, *NMs have the responsibility to conduct or implement ethics research*. NMs should support research [2, 4], set standards for research as well as disseminate and use research results [3]. Fourthly, *NMs have the responsibility to identify ethical problems* [12, 13]. Thus, NMs should be ethically sensitive. Ethical sensitivity is an attribute of ethically active NMs, it enables the identification of the ethical problems and emotional perceptions of vulnerable situations of others, as well as awareness of ethical outcomes of decisions made by others [14]. Ethically sensitive managers are able to make morally excellent and optimal decisions [15]. Finally, *NMs have the responsibility to solve ethical problems* [4, 12, 13, 16]. Thus, NMs should have the moral courage to defend and act on the values and principles of professional ethics and related laws, despite resistance by others or any adverse consequences to themselves [17]. Properly dealt with ethical problems promote organizations’ ethical culture [10].

All five above-mentioned dimensions of the ethical activity profile require different types of ethical activities from NMs, are equally important, and can be summarized together. To have a high ethical activity profile, NMs have to perform activities from all dimensions. However, based on earlier studies, only a limited number of NMs develop their own ethics knowledge [1, 12], influence ethical issues, conduct or implement ethics research [1]. Offering support for NMs can strengthen their ethical activity profile in the future. Support can be different kinds of ethical interventions [18] or development programs [19], including educational programs and training [6, 12, 15, 16].

Earlier studies point out that NMs are in need but lack support for ethics issues from their superiors and organizations [20]. Currently, health care organizations provide suboptimal levels of support in terms of multidisciplinary discussion of ethical issues, ethics education, and dealing with ethical issues [18]. Furthermore, some NMs desire more guidance toward becoming an ethical nurse manager [21], and becoming aware of, and carrying out their ethical activities [16]. Thus, there is an urgent need to support NMs’ contributions as ethical managers [20, 22]. In the field of health care and nursing ethics, there are very few earlier ethics intervention studies in general [23] and especially for NMs [4, 6]. Furthermore, ethics e-learning possibilities still seem to be lacking, even though e-learning has become mainstream in education and has undeniable significance [24] by being able to reach large numbers of potential participants at relatively low cost [25]. Thus, a new ethics educational e-learning intervention, Ethics Quarter (EQ), including all five dimensions of ethical activity profile of NMs, has been developed for research purposes at the University of Turku to support NMs.

Developing of an intervention is a systematic process consisting of several phases and components. When developing new interventions, scientific user evaluations are crucial [26] and thus, in this study, the user perspective usability, feasibility and fidelity of the EQ intervention have been evaluated. All three have been identified as essential criteria for the evaluation of e-learning interventions in health care since poor usability, feasibility or fidelity can influence the overall effectiveness of the intervention [25, 27]. In this study, usability is defined as the extent to which the EQ can be used by NM users to achieve high ethical activity profile with regard to effectiveness, efficiency, and satisfaction [28]. Feasibility is defined as the extent to which the EQ is possible and practical to perform conveniently [29].

Fidelity analysis, why or how the intervention works, could be included in each stage of the EQ intervention: design, delivery, receipt and enactment [30]. However, in e-learning interventions, where the user has a great deal of freedom to determine how to use the intervention and where interventions are reliant on retaining participants, a priority is seen in receipt, i.e. the extent to which participants actively use intervention materials [31] or in engagement. i.e. the level of participation or involvement, focusing on temporal patterns (e.g. duration) of use. Fidelity is defined in this study as engagement, and level of participation as duration of use of the EQ [32].

## Methods

### Aim of the study

This study evaluated the developed ethics educational e-learning intervention, the Ethics Quarter, from user perspective. The purpose was to evaluate the usability, feasibility and fidelity of the intervention, tested for the first time in this study in clinical environment after a randomized controlled trial (results of the effectiveness of the intervention have been reported elsewhere) [33]. The ultimate goal was to create a new way to support NMs’ ethical activity profile. The following research question was addressed: What is the usability, feasibility and fidelity of the Ethics Quarter intervention assessed by nurse managers?

### Design

A descriptive, cross-sectional design was used to evaluate the usability, feasibility and fidelity of the EQ intervention.

### Ethics quarter- intervention

The EQ educational e-learning intervention for NMs was developed according to the criteria for complex interventions in the health care (CReDECI2) guideline [34]. Based on literature and the construct of the ethical activity profile of NMs [1], the EQ was developed through several group discussions and feedback rounds by a multidisciplinary design team of nurse teachers, nursing management ethics researchers and NMs in 2018. Furthermore, two different expert panels consisted of a total of 10 (expert panel I) and 12 (expert panel II) postgraduate students, professors and ethics researchers at the department of Nursing Science in the University of Turku evaluated the structure of the EQ intervention. In spring 2019, a paper version of the EQ was translated into electronic version; an IT specialist coded the virtual learning environment. The prototype was tested by nurse managers (*n*=2) who used the EQ as planned and thus, no changes were made. In addition, the usability and feasibility of the EQ was pilot-tested (*n*=8): all master and postgraduate students of the Department of Nursing Science at the University of Turku working as nurse managers were invited to participate. Based on the pilot study results, an educational video about using the EQ educational area was added and some language corrections were made.

**Fig. 1:**
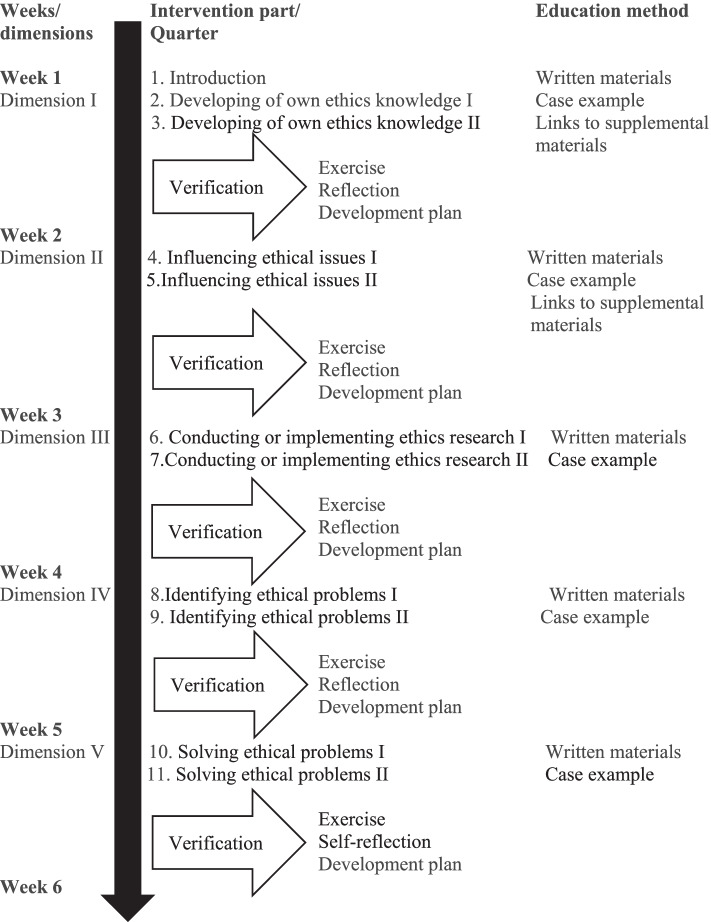
Timeline and components of the Ethics Quarter intervention

During the development phases, the structure of the EQ was clarified; two quarters comprise one dimension of NMs’ ethical activity profile. The EQ has five dimensions, 12 evidence-based educational quarters, including introduction and summary (Fig. 1). Both content and structure (Table 1) share the five dimensions of the ethical activity profile of NMs. In the EQ intervention, NMs have two quarters, one dimension per week, and they are able to participate at any time, in any place, the only requirement being access to the internet. The quarters are text material, including real-life role model experiences of the presented issue, e.g., NMs’ moral courage. Using case studies to highlight ethical leadership in the organization may be one way to explicitly bring learning of ethical role models to a wider group of managers [7].

**Table 1:** The content of the Ethics Quarter (EQ)

Intervention quarter	Description	Main references for the content
1. Introduction	What are the ethical activities of nurse managers and why are they important? Examples of failed activities. Structure of the EQ.	[2–4, 6, 7, 19–21]
2. Developing of own ethics knowledge I	Why develop one’s own ethics knowledge? What is ethics in nursing management? Why do nurse managers need ethics? What kind is ethical nurse manager? Ethics, values, laws, ethical guidelines, own ethical reflection.	
3. Developing of own ethics knowledge II	How is ethics knowledge developed? What are the ethical guidelines, ethical principles and codes of ethics of nurse managers? How can they be used?	
4. Influencing ethical issues I	Why and how can ethical issues be influenced? How can one increase ethical activities in an organization? How can one create an ethical organization?	[3–8, 10, 11, 20]
5. Influencing ethical issues II	How to recognize the threat of unethical action? How to operate in unethical issues? How to report ethically challenging situations? Reporting channels.	
6. Conducting or implementing ethics research I	Why should nurse managers conduct ethics research and implement ethics research? How can it be done?	[2, 4, 5]
7. Conducting or implementing ethics research II	How can values and ethical principles be defined and implemented into nurse managers’ own work unit?	
8. Identifying ethical problems I	What is an ethical problem? How can an ethical problem be identified? What kind of ethical problems do nurse managers have?	[12–15]
9. Identifying ethical problems II	Why identify an ethical problem? What is ethical sensitivity? Why should nurse managers be ethically sensitive? How can ethical sensitivity be strengthened?	
10. Solving ethical problems I	Why solve an ethical problem? What is moral courage?	[4, 10, 12, 13, 16, 17]
11. Solving ethical problems II	How can an ethical problem be solved? The ethical problem-solving process. Different kinds of activities to solve ethical problems.	
12. Summary	Review of the course	

The pedagogical basis of the EQ is constructivism. Principles of constructivism have been successfully adapted for previous e-learning [35], and constructivism does not prescribe rigid rules for designing a learning environment [36]. In this study, NMs are seen as active knowledge constructors who actively create their own subjective representations of the e-content. NMs progress in acquiring knowledge by themselves, one dimension per week, and new knowledge is linked to their prior knowledge and experiences [37]. After every dimension, NMs have to do an exercise entailing self-reflection and development plans, a total of five times. This provides them the opportunity to link their everyday experiences to ethical theory and actively construct knowledge and use their previous experiences. This multimethod intervention thus allows NMs’ self-reflection [23] and combining theory and practice [38].

### Participants and setting

Evaluation of the EQ was carried out by Finnish NMs (*n* = 95) after participating in the EQ intervention among the intervention group of a randomized controlled trial in 2020. The study is registered in ClinicalTrials.gov with the identifier: 04234503. NMs were recruited via the membership register of the Union of Health and Social Care Professionals in Finland (Tehy) trade union (https://www.tehy.fi/en) by e-mail. The union is a national professional interest group for registered nurses, NMs and advanced consultants/specialists in the social and healthcare sector who have a master’s degree (nursing science or related) or registered nurse’s degree. Inclusion criteria for the participants were: current work as a nurse manager and a sufficient command of the Finnish language. The introductory information e-mail included a short description of the study and the intervention and the link to the http://etiikanvartti.fi/?tutkimus website. The website featured complete information about the study; if the NM wanted to participate, s/he gave informed consent and filled in all the study measurements, including background factors.

For the main trial, 169 NMs were randomly allocated to the intervention group and received a password to log in to the learning environment. However, 50 users (29.5%) never signed in. The NMs who signed in (*n*=119) had very strong commitment to the EQ: (*n*=95) 79.8% completed it and the final response rate was 56.2% (95/169). The baseline background factors (*n*=169) were statistically compared between the NMs who 1) completed the EQ and participated in measurements (*n*=95), 2) got the password, but did not sign in to the EQ (*n*=50), and 3) signed in, but did not participate in measurements (*n*=24) (Table 2). NMs who completed the EQ and participated in measurements were statistically significantly older (*p*<0.005) than those who received the password, but did not sign in to the EQ and those who signed in, but did not participate in measurements. There were no other statistically significant differences (all *p* >.005) in the demographic characteristics between the groups.

**Table 2:** Nurse manager participant demographics

Participant demographics ***N*** = 169	NMs who completed the EQ and participated in measurements ***N*** = 95	NMs who got the password, but did not sign in to the EQ, ***N*** = 50	NMs who signed in, but did not participate in measurements ***N*** = 24	***p***-value
Variables	n (%)			
Age				.005**
Years, median (range)	52 (34-64)	48 (29-63)	47 (28-60)	
< 40	10 (10.5)	16 (32)	6 (25)	
40-49	28 (29.5)	12 (24)	9 (37.5)	
50-59	48 (50.5)	17 (34)	7 (29.2)	
≥ 60	9 (9.5)	5 (10)	2 (8.3)	
Highest education				.47
Registered nurse’s (or corresponding) degree	47 (49.5)	25 (50)	8 (33.3)	
Master’s degree (University of applied sciences)	31 (32.6)	14 (28)	8 (33.3)	
Master’s degree (University)	13 (13.7)	9 (18)	5 (20.8)	
Licentiate degree/ Doctoral degree (University)	0.00	0 (0)	1 (4.2)	
Other	4 (4.2)	2 (4)	2 (8.4)	
Employment sector				.41
Public	72 (75.8)	34 (68)	18 (75)	
Private	22 (23.2)	16 (32)	5 (20.8)	
Trust	1 (1.0)	0 (0)	1 (4.2)	
Position in organization				.18
Unit-level management	84 (88.4)	43 (87.8)	16 (69.6)	
Middle management	7 (7.4)	4 (8.2)	5 (21.7)	
Strategic management	4 (4.2)	2 (4.0)	2 (8.7)	
Length of work experience				.42
Years, median (range)	9 (0-37)	7 (0-34)	5 (0-30)	
< 5	24 (25.3)	19 (38)	10 (41.7)	
5-10	32 (33.6)	16 (32)	6 (25)	
> 10	39 (41.1)	16 (30)	8 (33.3)	
Number of subordinates				.99
Amount, median (range)	26 (0-260)	30 (3-150)	27 (7-500)	
< 21	31 (32.6)	18 (36)	7 (29.2)	
21-50	47 (49.5)	24 (48)	13 (54.1)	
51-100	12 (12.6)	6 (12)	0 (0)	
> 100	5 (5.3)	2 (4)	4 (16.7)	
Participation in continuing ethical education				.32
Yes	19 (20.0)	5 (10)	4 (16.7)	
No	76 (80.0)	45 (90)	20 (83.3)	
Participation in an ethical working group/committee				.11
Yes	7 (7.4)	3 (6.1)	5 (20.8)	
No	88 (92.6)	46 (93.9)	19 (79.2)	
Having an official ethics-related post				.09
Yes	8 (8.4)	0 (0)	1 (4.2)	
No	87 (91.6)	49 (100)	23 (95.8)	
Participating in an ethics research project				
Yes	0 (0)	0 (0)	0 (0)	
No	95 (100)	49 (100)	23 (100)	
Participating in an ethics development project				.21
Yes	2 (2.1)	1 (2)	2 (8.7)	
No	93 (97.9)	49 (98)	21 (91.3)	
Having an ethics organizational structure				.71
Yes	23 (24.2)	15 (30)	7 (29.2)	
No	72 (75.8)	35 (70)	17 (70.8)	

Categorical variables tested with Fisher’s exact, continuous with Kruskal-Wallis test. *NM* nurse manager. *EQ* Ethics Quarter. **p < .01

### Procedures

The EQ intervention was standardized by using the same login process, EQ page and materials for all the participants. The proceeding possibilities during the intervention were also similar to all in the virtual learning environment: The first dimension of the intervention was open at the starting point and the second and all subsequent dimensions opened automatically, one dimension per week, of which the participants were reminded by e-mail based on the timestamp of the user rights creation moment. There were some internal barriers and facilitators which potentially influenced the delivery of the intervention: in order to produce learning, the intervention lasted 6 weeks, and thus required high engagement. However, to complete one quarter took about 15 min and it was possible to carry it out on a tablet, laptop or smartphone.

### Data collection and outcome instruments

The usability, feasibility and fidelity of the EQ were evaluated right after NMs completed the whole intervention, after 6 weeks. The usability was measured with the System Usability Scale (SUS) [28]; Finnish version, [39]. The SUS is a 5-point Likert scale (1=totally disagree; 5=totally agree) featuring 10 items, e.g. “I found the website to be simple” and “I think that I could use the website without the support of a technical person”. The SUS provides a single reference score ranging from 0 to 100 (worst-to-best) and the average SUS score is 68 [40], with higher scores indicating higher usability. The SUS is valid, reliable and adequate for measuring general usability [28, 40].

Feasibility was measured with structured independent 5-point Likert (1=totally disagree; 5=totally agree) questions, created for this study, concerning the EQ as a learning method, EQ duration and EQ contents (See questions in Table 3), and with one open question: “Your open feedback about content of the EQ?” (Table 4). Questions were based on literature and face validity of the instruments was assessed by postgraduate students, professors and ethics researchers in nursing science (expert panel I, n=10) to provide insight into how potential participants might interpret and respond to the items. Data were collected using REDCap electronic data capture tools hosted by the university. The REDCap (Research Electronic Data Capture) is a secure, web-based software platform designed to support data capture for research studies [41].

**Table 3:** Usability scores, System Usability Scale, and feasibility scores of the Ethics Quarter website (*n* = 93-95)

**Usability scores, System Usability Scale, ranging from 0 to 4.**	***n***	**Mean**	**SD**	**Min**	**Max**
1. I think that I would like to use this website frequently.	95	3.22	0.70	1	4
2. I found the website to be simple.	95	3.38	0.73	1	4
3. I thought the website was easy to use.	95	3.42	0.85	0	4
4. I think that I could use the website without the support of a technical person.	95	3.74	0.61	1	4
5. I found the various functions in this website were well integrated.	95	3.39	0.85	1	4
6. I thought there was a lot of consistency in this website.	95	3.45	0.68	1	4
7. I would imagine that most people would learn to use this website very quickly.	95	3.55	0.56	2	4
8. I found the website very intuitive.	95	3.16	0.84	0	4
9. I felt very confident using the website.	94	3.23	0.87	1	4
10. I could use the website without having to learn anything new.	94	3.65	0.60	1	4
**The SUS sum score**	**94**	**85.40**	**14.27**	**42.50**	**100.00**
**Feasibility scores ranging from 1 to 5**	**n**	**Mean**	**SD**	**Min**	**Max**
1. The EQ offered a good way to learn nursing management ethics	95	4.65	0.50	3	5
2. The duration of the EQ was adequate.	93	4.62	0.51	3	5
3. The contents of the EQ were interesting.	95	4.68	0.51	3	5
4. The contents of the EQ increased my knowledge of nursing management ethics.	93	4.59	0.63	2	5
5. The contents of the EQ were sufficiently challenging.	95	4.15	0.81	2	5

*SD *Standard Deviation

*EQ *Ethics Quarter

**Table 4:** Examples of open question content feedbacks about the Ethics Quarter, *n* = 71

**Positive feedback,*n*** **= 70**	
“*I found the education to be extremely good and necessary. It led me to think about ethical questions more deeply, to pay attention to ethical challenges also in a very ordinary basics and indeed I felt…like I was getting confirmation and certainty concerning ethical decision-making and more courage into it.*” (13)	
*“In my opinion, this was a really good online course. I would allow all nurse managers, at every level, to do this.”* (28)	
*“A lot of time was saved, by our not needing to travel to receive this education; we were allowed to consume it when we had time.”* (67)	
**Negative feedback,*n*** **= 1**	
*“Dividing this education into small pieces made it somehow scattered. In my opinion, it covered a bit too many self-evident issues. There could have been more guidance into reflection.”* (56)	
**Developmental feedback,*n*** **= 8**	
“*There could have been some more challenging ethical problems to solve and possibly also other respondents’ responses to look over (anonymously, of course).”* (6)	
“*As a mouth healthcare professional and nurse manager, I would have hoped for case examples on a wider stage. In all health care education, they generalize nursing into nurses’ work or work related to general medicine.*” (70)	

Fidelity was evaluated with the EQ web-page user information provided by Google Analytics. The evaluation was based on how much time [31, 32] participants spent engaging with the programme. NMs used for one dimension (two quarters) of the intervention according to URL addresses.

The background factors asked were age, highest education, employment sector (public, private or trust), position in organization, length of work experience, number of subordinates, participation in continuing ethical education, participation in an ethical work group or committee, having an official ethics-related post, participating in an ethics research project, participating in an ethics development project, and having some kind of ethics organizational structure in work organization, e.g., a clinical ethics committee (Table 2).

### Data analysis

In the usability evaluation, the SUS items were scored before the analysis according to the Brooke system [40], leading to item contributions ranging from 0 to 4 (from the most negative to the most positive response). The mean SUS score and SUS item score comparisons between the demographic variables were conducted by using the Kruskal-Wallis test and continued with Steel-Dwass for multiple comparisons. Analyses were performed using the SAS version 9.4 for Windows software (SAS® Institute Inc., Cary, NC, USA). The level of statistical significance was set at *p*-value 0.05 (two-tailed).

In the feasibility evaluation, similar statistical analyses as above were conducted for feasibility item scores. Furthermore, inductive content analysis [42] and quantification were used to analyze the one open question concerning feasibility. The transcribed version of the source material was typed out, resulting in seven A4 pages. The focus of the qualitative analysis was the feedback about the content of the EQ. The units of analysis were the responses to the question: What is the feedback of the content of the Ethics Quarter as assessed by nurse managers? A meaning unit was used, namely, a constellation of words concerning the feedback of the content. Meaning units were divided into three categories – positive, negative and developmental feedback – by the main researcher. The quantitative analysis focused on the number of negative, positive and constructive comments to highlight the amount of positive, negative and developmental comments.

In the fidelity evaluation, it was calculated how much time on average NMs spent on one dimension (two quarters). The intervention was planned to take 15 minutes per quarter, 30 minutes per dimension; this was the basis for comparison.

### Ethical considerations

The study was approved by the University Ethics Committee in February 2020 and by the (Tehy) trade union in January 2020. Responsible conduct of research [43] was followed throughout all study phases. The NMs received written information about the purpose of the study and the option to withdraw at any point. All NMs gave their informed consent before entering the study. The EQ learning environment was password-protected to guarantee participant confidentiality. The research data was collected only in REDCap and thus, stored on a secure platform.

## Results

The usability of the EQ was reportedly high [40]. The mean SUS sum score was 85.40 (Standard deviation, SD 14.27, range 42.50–100.00) (Table 3). The perception of being capable to use the app without the support of a technical person (item 4) showed the highest mean score (3.74, SD 0.61). No background factors were statistically significantly associated with the mean SUS score or single items.The feasibility of the EQ was reportedly high (Table 3); all the mean values were above 4 (range 1–5). No background factors were statistically significantly associated with single feasibility items. The NMs considered that the EQ offered a good way to learn nursing management ethics, the mean score being 4.65 (SD 0.50, range 3–5). The duration of the EQ was reportedly adequate; the mean score was 4.62 (SD 0.51, range 3–5).

Open feedback about content of EQ was obtained in 75% (*n* = 71) of the 95 responses. The responses were positive in 99% (*n* = 70) of cases and negative in 1% (*n* = 1). Of the responses, 11% (*n* = 8) also included development propositions. To summarize, positive feedback considered that the EQ contents were good, necessary, time-saving, practical and research knowledge-based, suitable for all nurse managers to perform. Negative feedback considered the contents self-evident and too scattered. Developmental feedback suggested that contents should be more challenging and interactive and more exercise contents were hoped for (Table 4).

NMs reported that the contents of the EQ were interesting; the mean score was 4.68 (SD 0.51, range 3–5): “*A lot of new and usable knowledge”* (29), *“The contents were based on research knowledge”* (30), “*Contents were clear and vigorous. The issues were summarized very well into an understandable and plain language format. I could have studied more ethics this way*.” Managers considered that the contents of the EQ increased their knowledge in nursing management ethics; the mean score was 4.59 (SD 0.63, range 2–5). NMs described the increased knowledge as follows: *“Really good study set and so clear that it increased my own understanding of ethics issues”* (86), *“Good content, gave me tools to implement ethical activities into practice”* (46), *“I got a lot of where to lean on”* (50), *“The course got me thinking more about ethical issues and to take them into consideration; I believe it’s also going to continue in the future”* (69). (58). The NMs considered the contents of the EQ to be sufficiently challenging, 4.15 (SD 0.81, range 2–5): “*The contents were sufficient in relation to the time reserved”* (21), *“the exercises led me to reflect on the issue more accurately”* (139).

The fidelity of the EQ was average based on the time used for one dimension. According to Google Analytics, NMs spent on average 18.57 minutes to complete one dimension.

## Discussion

This study produced valuable user perspective evaluation of the new ethics e-learning intervention. The EQ intervention was tested for the first time in a clinical environment for nurse managers and evaluated from the perspective of usability, feasibility and fidelity. Managers perceived the feasibility and usability of the EQ intervention as good: the EQ offered a feasible and usable way to support their ethical activity profile. Furthermore, the fidelity was average. There are only a few earlier ethics interventions for NMs even though previous literature has pointed out NMs’ needs to be supported in ethics issues [16, 18, 20, 21]. Thus, the developed and user-evaluated EQ intervention is valuable for NMs.

There is little earlier empirical knowledge on how to impart ethical skills to NMs in practice [4, 6]. In the recent systematic review of Ravaghi et al. [44], focusing on studies that develop and strengthen the competencies and skills of health care managers covering the years 1990–2019, researchers found no studies including ethics (as taught skills and expected outcomes). Our findings offer new empirical knowledge on how to educate NMs for ethical activities. We have described the intervention structure, contents, timeline and process, allowing others to implement it. Furthermore, the EQ offers basic education based on the latest information and materials [45] about values, ethics, ethical management and ethical activities for NMs and aims to answer to the evidence of Devik et al., [16]: NMs require greater awareness and understanding of what ethical leadership means. In this evaluation study, NMs stated that the EQ contents increased their knowledge of nursing management ethics and that the EQ offered a good way to learn nursing management ethics.

In this study, the e-learning method was considered to be a feasible, possible and practical way of learning ethics and ethical activities conveniently [29], and NMs would also like to use it in the future. In earlier studies of other management competencies, NMs have similarly considered e-learning to be a suitable, modern [44] and supportive [6] method of learning. In this study, NMs participated in the EQ nationwide and they could not be brought together for education simultaneously in one place. Thus, e-learning offered a sustainable solution, as recommended by Abel, Hall, Swartz & Madigan [46].

The EQ intervention’s developmental areas were also identified in this study. The time used by the NMs in the educational area was shorter than estimated. On average, NMs used only 18.57 minutes per dimension, not 30 minutes as planned. According to a previous Cochrane review, it seems that healthcare professionals have quite low engagement with e-learning interventions [47]. Furthermore, some managers indicated in the feasibility questions that the contents of the EQ could have been even more challenging and there was quite high standard deviation in answers to the question of whether the contents of the EQ increased NMs’ knowledge of nursing management ethics. Thus, in the future, even more challenging contents in the EQ could possibly be tested. Furthermore, NMs indicated the wish to see other respondents’ responses in developmental feedback. It is known that social interaction plays a role in learning [35] and thus, possibilities of including interactivity in the EQ should be evaluated in the future.

The EQ intervention seems to be valuable for all health care organizations, which currently provide suboptimal levels of support for ethics for NMs [16, 18]. Also in this study, only 24 percent of the participating NMs’ organizations had some kind of organizational ethics structure. The EQ could be taken systematically into use in healthcare organizations. It must be noted that the influences of ethical activities occur not only directly, among immediate followers within a unit, but also indirectly, across hierarchical levels. Senior managers can facilitate the effects of subordinate managers’ ethical activities; thus it is important to ensure development of senior managers to exhibit high levels of ethical leadership [48]. Therefore, education should start with those at higher levels of management, followed by unit-level managers. Most of the NMs in this study were in charge of the unit and in open responses, they also recognized that all the levels of management should be educated. Furthermore, one of the main challenges identified in manager education relates to the fact that managers work in teams but are often trained as individuals [45], even if it may be more effective if the whole management team can be trained simultaneously.To increase ethical activities, using different approaches within the same institution is preferred [49]. Thus, after EQ, organizations could continue ethics work monthly with different continuing approaches, such as multi-professional committees for consultation and discussion of ethical issues [12] or ethics reflections. Short, daily reflections among the work team at the end of every day could also be an option. Doing something, even if it is just bringing up ethical issues regularly during staff meetings, is far better than doing nothing at all [9].

### Strengths and limitations

The strength of this study lies in its evidence-based intervention development: by following the (CReDECI2) guideline criteria [34] we aimed to develop, together with the end-users, NMs, a usable, feasible and actively used intervention. Furthermore, the proper description of the EQ intervention allows its evaluation and future replication. This study also provided a justified evaluation of the feasibility and usability of EQ by a large nationwide group of EQ end-users, NMs at different levels of healthcare organizations, and they also provided feedback data for the next phases of the program’s further development. Additionally, NMs’ commitment to the EQ was high, suggesting that the intervention was greatly needed, while the burden it caused in the complex, continuously changing environment was reasonable.

This study also has some limitations. Feasibility was measured with independent structured questions created for this study, never validated; thus, conclusions have been made with caution and strengthened by open question responses. The fidelity of this study was measured only relying on Google Analytics statistics. A multi-perspective, comprehensive multi-method approach would have offered better insight into fidelity, e.g. into enactment [30]: did NMs perform the ethical activities they learned during the intervention afterwards? Furthermore, by focusing the study also on the two other participant groups (those who never signed in and those who signed in but did not participate in measurements), some key engagement information about the intervention more generally could have been obtained. Also, lack of a proper process evaluation is a clear limitation; it could have offered valuable insight into how the EQ could be optimized even more [26].

## Conclusions and future directions

Our findings suggest that an ethics educational e-learning intervention, the Ethics Quarter, is a usable, feasible and not too burdensome option for supporting NMs’ ethical activity profile in health care organizations. The results of this user perspective study advance the understanding about educating NMs in ethics issues with the e-learning method and the main concern seems to be lack of engagement. Thus, in future studies, easily added, more extensive user logs could give insight into what elements of e-learning interventions increase the level of participation or make NMs return to the e-learning environment. Furthermore, it would be crucial to investigate enactment, i.e. whether the strengthened ethical activity profile of NMs also translates into actual ethical activities of NMs in healthcare organizations. A further step in developing the EQ intervention itself would be increasing the challenge of the EQ and implementing some interactivity into the educational area; for example, through common discussions or other participants’ readable responses as a possible option. The contents of the intervention must also be constantly updated based on the latest knowledge of ethical activities. Furthermore, contents of the EQ could be revised in the future for the whole multi-professional management team.

## Data Availability

Due to the sensitive nature of the questions asked in this study, survey respondents were assured raw data would remain confidential and would not be shared, hence, data are not available as it is confidential.

## Abbreviations

EQ: Ethics Quarter
NM: Nurse manager
